# A 2D-DIGE-based proteomic analysis brings new insights into cellular responses of *Pseudomonas putida* KT2440 during polyhydroxyalkanoates synthesis

**DOI:** 10.1186/s12934-019-1146-5

**Published:** 2019-05-28

**Authors:** Justyna Możejko-Ciesielska, Agnieszka Mostek

**Affiliations:** 1 Department of Microbiology and Mycology, Faculty of Biology and Biotechnology, University of Warmia and Mazury in Olsztyn, Oczapowskiego 1A, 10-719 Olsztyn, Poland; 2 Department of Gamete and Embryo Biology, Institute of Animal Reproduction and Food Research, Polish Academy of Sciences in Olsztyn, Tuwima 10, Olsztyn, Poland

**Keywords:** PHAs, *Pseudomonas putida* KT2440, Proteomics, Response

## Abstract

**Background:**

Polyhydroxyalkanoates (PHAs) have attracted much attention in recent years as natural alternatives to petroleum-based synthetic polymers that can be broadly used in many applications. *Pseudomonas putida* KT2440 is a metabolically versatile microorganism that is able to synthesize medium-chain-length PHAs (mcl-PHAs). The phenomena that drive mcl-PHAs synthesis and accumulation seems to be complex and are still poorly understood. Therefore, here we determine new insights into cellular responses of *Pseudomonas putida* KT2440 during biopolymers production using two-dimensional difference gel-electrophoresis (2D-DIGE) followed by MALDI TOF/TOF mass spectrometry.

**Results:**

The maximum mcl-PHAs content in *Pseudomonas putida* KT2440 cells was 24% of cell dry weight (CDW) and was triggered by nitrogen depletion. Proteomic analysis allowed the detection of 150 and 131 protein spots differentially regulated at 24 h and 48 h relative to the cell growth stage (8 h), respectively. From those, we successfully identified 84 proteins that had altered expression at 24 h and 74 proteins at 48 h of the mcl-PHAs synthesis process. The protein–protein interactions network indicated that the majority of identified proteins were functionally linkage. The abundance of proteins involved in carbon metabolism were significantly decreased at 24 h and 48 h of the cultivations. Moreover, proteins associated with ATP synthesis were up-regulated suggesting that the enhanced energy metabolism was necessary for the mcl-PHAs accumulation. Furthermore, the induction of proteins involved in nitrogen metabolism, ribosome synthesis and transport was observed. Our results indicate that mcl-PHAs accumulated in the bacterial cells changed the protein abundance involved in stress response and cellular homeostasis.

**Conclusions:**

The presented data allow us to investigate time-course proteome rearrangement in response to nitrogen limitation and biopolyesters accumulation. Our results have pointed out novel proteins that might take part in cellular responses of mcl-PHA-accumulated bacteria. The study provides an additional knowledge that could be helpful to improve the efficiency of the bioprocess and make it more economically feasible.

**Electronic supplementary material:**

The online version of this article (10.1186/s12934-019-1146-5) contains supplementary material, which is available to authorized users.

## Background

Polyhydroxyalkanoates (PHAs) are a class of biodegradable polyesters accumulated intracellulary by a wide range of bacteria in the form of granules that serve as a storage material for energy. Due to the biodegradability, biocompatibility and thermoplasticity these biopolymers are considered as a substitute for synthetic polymers. It is known that nutrient starvation increases PHAs productivity depending on the bacterial species and the type of substrate that is used as a carbon source. The first PHA [poly (3-hydroxybutyrate)] was found in *Bacillus megaterium* [1]. Later, the discovery of poly(3-hydroxyoctanoate) in *Pseudomonas oleovorans* proved that many types of PHAs could be synthesized by bacteria [2]. Polyhydroxyalkanoates are divided into two groups: short-chain-length (scl-PHA) containing 3–5 carbon atoms and medium-chain-length (mcl-PHA) consisting of more than 6 carbon atoms. Especially, mcl-PHAs have gained much attention in recent years because of more favourable properties than scl-PHAs making them great potential in many industrial applications. Generally, *Pseudomonas* species possess the gene clusters that determine the ability to synthesize and accumulate mcl-PHAs. The key genes involved in the mcl-PHAs biosynthesis are known, however there is a lack of information about the regulation mechanisms that are responsible for this process. The metabolism of polyhydroxyalkanoates synthesis has been extensively studied. Since the publication of *Pseudomonas putida* KT2440 complete genome, it has been considered as a model microorganism for genetic studies [3, 4]. It allowed to analyze the ability of this bacterium to survive under stressful conditions and to synthesize biopolyesters, our knowledge about its metabolism at the molecular level has increased significantly. However, the regulatory mechanisms that drive PHAs synthesis and accumulation are complex and still poorly understood. It is known that PHAs biosynthesis is related with central pathways such as fatty acid β-oxidation and de novo fatty acid synthesis. Furthermore, PHAs metabolism is encoded by the *pha* cluster and may be driven by the network of local and global regulators controlling the pathways involved in carbon and nitrogen assimilation [5]. However, it is not yet clear how *Pseudomonas putida* KT2440 re-arranges its whole metabolism under nutrients starvation and the excess of related substrates, resulting in a high level of PHAs synthesis and accumulation. Microbial proteomics has become a useful tool to determine new functions of gene products as it represents not only the gene product but additionally translational rate and post-translational modifications. Two-dimensional difference gel electrophoresis (2D-DIGE) is a technique for use in quantitative approaches, allowing the separation of proteins in different samples on the same gel and eliminating gel-to-gel variability. Identification of proteins that abundance is changed could gain insights into the mechanisms of PHAs biosynthesis under certain environmental conditions. The use of an advanced proteomic technique such as 2D-DIGE holds promise for the elucidation of the association between PHAs synthesis and nutrient starvation. However, 2D-DIGE technique has not yet been used in the research on PHAs. To the best of our knowledge, only in three publications the mcl-PHAs biosynthesis process was analyzed using proteomics approach. Poblete-Castro et al. [6] analyzed the metabolic response of *Pseudomonas putida* KT2442 cultured in sodium decanoate to different nutrient-starvation strategies using 2D-PAGE analysis. Also, proteome of *Pseudomonas putida* LS46 [7] and *Pseudomonas putida* CA-3 [8] was evaluated, these bacteria were cultured on biodiesel-derived waste glycerol and styrene, respectively. The above mentioned reports revealed some information on mechanisms underlying PHA biosynthesis using non-related substrates. However, there is still a lack of studies that considered the cellular regulatory mechanisms leading to biopolyesters production from related carbon sources using time-course proteomic approach.

Therefore, the aims of the present study were to compare the proteome of *Pseudomonas putida* KT2440 at different time points of its cultivations using the 2D difference gel electrophoresis (2D-DIGE) technique and to identify differentially abundant proteins potentially involved in mcl-PHAs synthesis process.

## Results and discussion

### Cell growth and mcl-PHAs synthesis

**Fig. 1:**
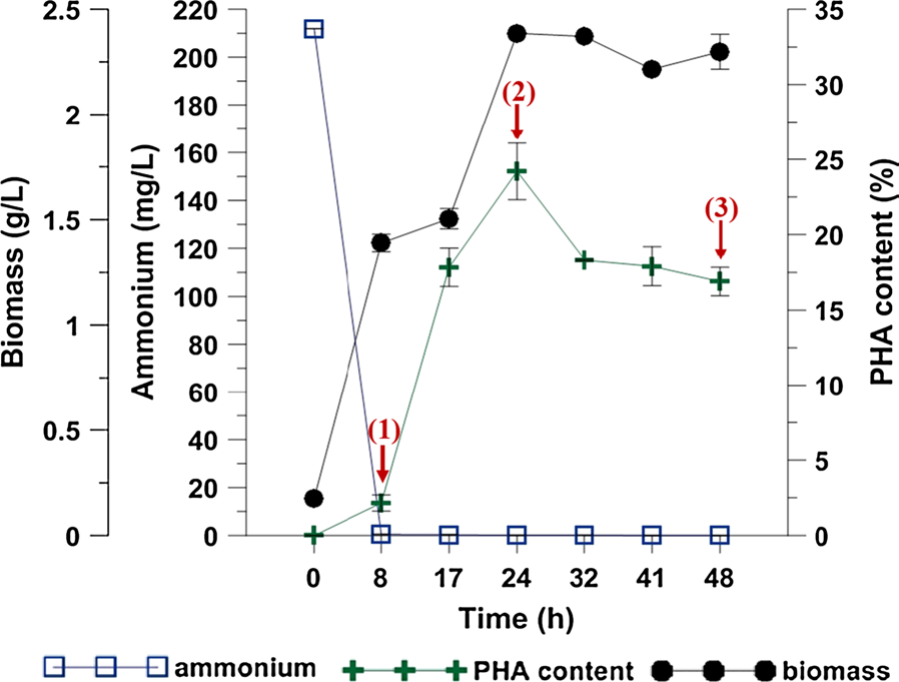
Mcl-PHAs content, biomass and ammonium concentration during cultivations of *Pseudomonas putida* KT2440. Numbers in parenthesis indicate the sampling time of biomass for proteomic analysis. (1), (2) and (3) indicate samples collected at 8 h, 24 h and 48 h of the cultivation, respectively

Biopolyesters synthesis is often investigated by comparing the nutrient-sufficient stage to nutrient-deficient stage. It was speculated that species belonging to *Pseudomonas* require nitrogen-deprived media for synthesizing PHAs from unrelated carbon sources but not from related substrates [9]. To determine the proteome response of *Pseudomonas putida* KT2440 to mcl-PHAs synthesis, the cultivation under nitrogen limitation was conducted. The fermentation process in a bioreactor enabled to separate mcl-PHAs synthesis phase from the growth phase and to monitor precisely the bioprocess due to larger volume for sampling. The maximum specific growth rate (μ_max_) of *Pseudomonas putida* KT2440 was found to be 0.68 h^−1^ (data not shown). Lower μ_max_ was observed when the same strain was cultivated on octanoate (0.26 h^−1^) and on citrate (0.54 h^−1^) [10]. The ammonium concentration, cell dry weight (CDW) and mcl-PHAs content in the analyzed strain during growth are shown on Fig. 1. The amount of mcl-PHAs increases under nitrogen starvation. Ammonium was completely consumed at 8 h of the fermentation. CDW increased up rapidly to 17 h, and then slowed down. Immediately, after ammonium exhaustion in the growth medium, mcl-PHAs concentration started to increase gradually up to 17 h of the fermentation process, and then slightly decreased. The maximum mcl-PHAs content in *Pseudomonas putida* KT2440 cells was 24% of CDW, with a corresponding PHA yield of 0.60 g/g and was triggered by nitrogen depletion. The biopolymer concentration and yield was higher than that reported by Follonier et al. [10] during growth of the same strain on sodium gluconate. The obtained results confirmed that the mcl-PHAs content was correlated with the biomass concentration.

The extracted and purified mcl-PHAs were determined by GC of the 3-hydroxyalkanoates methyl esters to determine their repeat-unit composition (Table 1). At 8 h of the bioprocess the analyzed strain showed a tendency to accumulate large amounts of 3-hydroxyhexanoate (3HD) and 3-hydroxydodecanoate (3HDD) and a trace amount of 3-hydroxyoctanoate (3HO). After this time-point, 3HD and 3HO were detected as the main constituents. Furthermore, a trace amount of 3-hydroxytetradecanoate (3HTD) was found in the extracted mcl-PHAs. The major repeat units were similar to those produced by other *Pseudomonas* species cultivated on related carbon sources [11].

**Table 1:** Monomeric composition of mcl-PHAs produced by *Pseudomonas putida* KT2440 in bioreactor experiments

Culture time (h)	Relative monomer composition of purified PHAs (% w/w)							
	3HHx	3HO	3HN	3HD	3HUD	3HDD	3HTD	3HHxD
8	n.d.	5.72 ± 0.3	n.d.	71.02 ± 1.3	n.d.	23.26 ± 0.7	n.d.	n.d.
17	1.24 ± 0.1	13.70 ± 0.8	n.d.	74.78 ± 0.9	n.d.	8.76 ± 0.6	1.52 ± 0.4	n.d.
24	1.37 ± 0.2	16.48 ± 0.9	n.d.	72.09 ± 0.7	n.d.	8.92 ± 0.2	1.15 ± 0.1	n.d.
32	1.18 ± 0.1	14.16 ± 0.7	n.d.	74.69 ± 0.7	n.d.	8.96 ± 0.3	1.01 ± 0.3	n.d.
41	1.34 ± 0.6	15.58 ± 0.9	n.d.	72.29 ± 0.8	n.d.	9.74 ± 0.4	1.05 ± 0.2	n.d.
48	1.23 ± 0.8	14.48 ± 0.7	n.d.	74.21 ± 1.5	n.d.	9.10 ± 0.8	0.98 ± 0.2	n.d.

Mean values are calculated from triplicate measurements

n.d., not detected, less than 0.3%; 3HHx, 3-hydroxyhexanoic acid; 3HO, 3-hydroxyoctanoic acid; 3HN, 3-hydroxynonanoic acid; 3HD, 3-hydroxydecanoic acid; 3HUD, 3-hydroxyundecanoic acid; 3HDD, 3-hydroksydodecanoic acid; 3HTD, 3-hydroxytetradecanoic acid; 3HHxD, 3-hydroxyhexadecanoic acid

### Overview of proteomic data

**Fig. 2:**
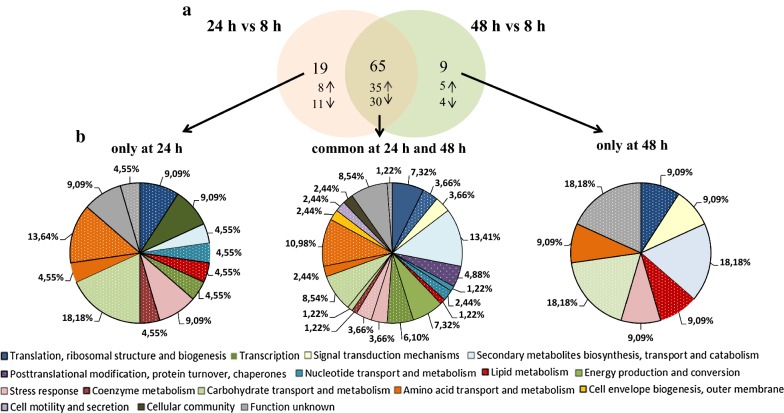
Classification of the identified proteins into role categories. **a** Venn diagram showing the number of proteins differentially expressed (fold change ≥ 2; p-value ≤ 0.05) at 24 h and 48 h of the mcl-PHAs synthesis bioprocess as compared to the *Pseudomonas putida* KT2440 growth stage (8 h). Three proteins set groups were created representing proteins which the abundance was changed only at 24 h, at both time-points and only at 48 h. **b** Functional classification of identified proteins according to their predicted and unknown functions. The pie charts illustrate the percentage of proteins in each group accounted for the total number of proteins with changed expression. The fields which illustrate the up-regulated proteins were dotted, whereas the field with down-regulated proteins were solid

**Fig. 3:**
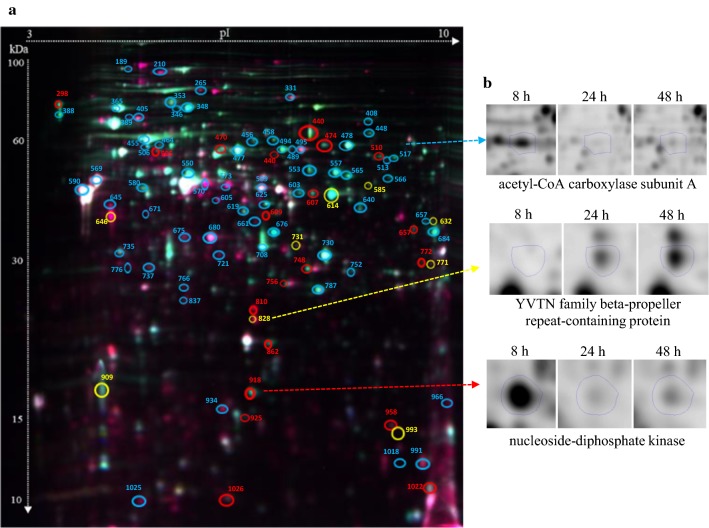
**a** Representative pattern of spots after 2D-DIGE analysis of *Pseudomonas putida* KT2440 proteome. Numbers indicate spots that significantly differ between 8, 24 and 48 h. Blue colour indicates proteins more abundant at 24 h and 48 h. Red colour indicates proteins in higher abundance only at 24 h and yellow colour indicates proteins more abundant only at 48 h. **b** Examples of normalized DIGE spots

**Fig. 4:**
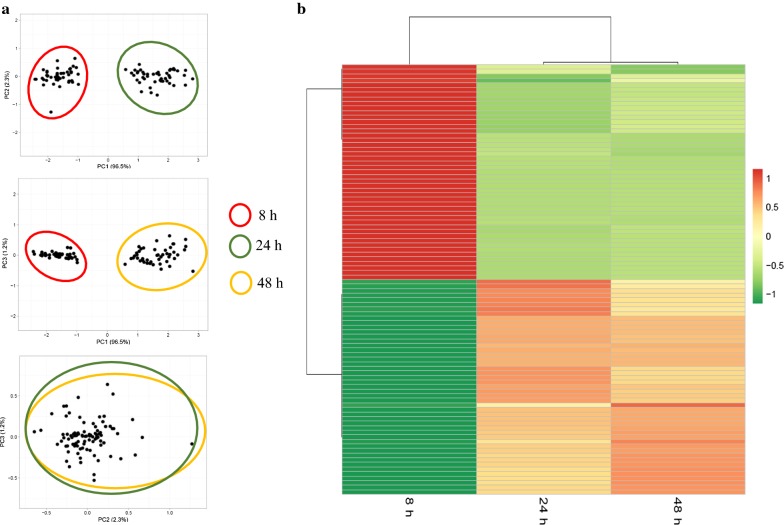
Protein abundance analysis in *Pseudomonas putida* KT2440 during mcl-PHAs synthesis. **a** PCA plots of the 2D-DIGE proteomics profile at 8 h, 24 h and 48 h of the bioprocess. **b** Cluster analysis of the normalized proteins spots abundance that were differentially regulated at 24 h and 48 h compared to 8 h

To investigate the proteomic response of *Pseudomonas putida* KT2440 to mcl-PHAs synthesis and accumulation process, the bacterial cells were harvested from the cultures at 8 h (a trace amount of mcl-PHAs), 24 h (fast mcl-PHAs accumulation) and 48 h (slow mcl-PHAs accumulation) for proteomic analysis (Fig. 1). Protein expression profile of the samples collected at 24 h and 48 h was compared to this at 8 h of the bioprocess to examine how protein expression pattern changed over time. Based on the analysis, 150 and 131 protein spots have a significantly altered expression at 24 h and 48 h (relative to 8 h of the growth), respectively. They were excised from gels and subjected to MALDI-TOF/TOF analysis. Using the Mascot search engine and the SwissProt database 84 proteins (43 up-regulated and 41 down-regulated) were successfully identified for 24 h of the biofermentation process, and 74 proteins (40 up-regulated and 34 down-regulated) for 48 h of the cultivation as compared to 8 h of the bacterial growth (Figs. 2a, 3; Additional file 1: Table S1). Since at both time-points mcl-PHAs were identified in *P. putida* KT2440 cells, a substantial amount of overlap in the protein expression patterns at 24 and 48 h had been expected. Indeed, 65 of the 84 proteins that had altered expression at 24 h as compared to the beginning of the culture were also differentially regulated at 48 h (Fig. 2a). In the set of 65 proteins, 35 of them were up-regulated and 30 were down-regulated. Some spots were represented by two proteoforms such as dihydrolipoamide dehydrogenase; acetyl-CoA carboxylase biotin, nitrogen regulatory protein P-II, adenosylhomocysteinase at 24 h of the cultivation and heat shock protein 90, chaperonin GroEL, isopropylmalate isomerase large subunit, elongation factor Tu-B, general amino acid ABC transporter periplasmic binding protein OmpF family protein at both analyzed time-points. It is worth to mention that the expression pattern analysis was also made between 24 and 48 h of the cultivation of *P. putida* KT2440. However, the differentially expressed proteins were not identified. Hierarchical cluster analysis presented as heat maps and principal component analysis (PCA) proved that the proteins profile from 24 and 48 h are more similar to each other than to 8 h of the bioprocess, but the results also showed substantial differences between 24 h versus 8 h and 48 h versus 8 h (Fig. 4a, b).

### Functional categories and protein–protein interaction analysis

Differentially expressed proteins were grouped using gene ontology annotation. Figure 2b presents an analysis of identified differentially expressed proteins at 24 h and 48 h of the bioprocess compared to 8 h of the cultivation. Generally, proteins involved in amino acid transport and metabolism (10.58%), carbohydrate transport and metabolism (8.54%), energy production and turnover (6.10%) were significantly induced at both time-points. Moreover, in this group, 13.41% of proteins related to secondary metabolites biosynthesis, transport and catabolism were down-regulated. As illustrated by pie chart, the majority of up-regulated proteins identified only at 24 h were connected with amino acid and carbohydrate transport and metabolism, translation, ribosomal structure and biogenesis. In addition, at 48 h of the cultivation, all proteins involved in amino acid biosynthesis, and signal transduction mechanisms were repressed compared to 8 h of the cultivation.

The identified proteins were also subjected to STRING neighborhood analysis (Additional file 2: Fig. S1). 63 proteins for 24 h and 58 proteins for 48 h were successfully recognized in the STRING database and represented as a network of proteins connected with confidence-based edges. The protein–protein interactions network demonstrated that the majority of identified proteins were connected to each other from medium to the highest interactions (score 0.4–0.9) indicating that the proteins are functionally linkage. At 24 h as well as 48 h of the cultivation, about 30% of identified differentially expressed proteins were unconnected. They were proteins mainly involved in peptidoglycan biosynthesis, transport, thiamine metabolism, regulatory processes and those classified as uncharacterized.

### Carbon and energy metabolism

During mcl-PHAs biosynthesis process several significant changes in the central energy pathways were observed. Due to the lack of 6-phosphofructokinase, *Pseudomonas putida* KT2440 has an incomplete glycolysis pathway. Thus, gluconate is utilized by these bacteria exclusively via the Entner-Doudoroff route [12]. Firstly, gluconate is transported to the cytoplasm by the action of GnuK transporter where it is converted to 6-phosphogluconate which enters the Entner-Doudoroff pathway. Then, 6-phosphogluconate dehydratase enzyme (Edd enzyme) transformed it into 2-keto-3-deoxy-6-phosphogluconate. The abundance of four proteins involved in substrate utilization, glyceraldehyde-3-phosphate dehydrogenase (spot 684), 6-phosphogluconolactonase (spot 752), phosphogluconate dehydrogenase (spot 409) and fructose-1,6-bisphosphate aldolase (spot 625), were significantly decreased at 24 h and 48 h of the cultivations relative to the 8 h of the mcl-PHAs biosynthesis. Furthermore, TCA cycle is also the main energy generator that produces GTP and the reducing power, the NADPH and FADH. In this study, the expression of proteins (succinyl-CoA ligase subunit beta, spot 603; succinyl-CoA synthetase subunit beta, spot 614; succinate-CoA ligase subunit alpha, spot 730) involved in TCA cycle were also down-regulated. The above-mentioned data could suggest that glycolytic pathway and TCA cycle are hampered upon nitrogen starvation which may lead to a greater carbon flux to acetyl-CoA synthesis, and thus increasing mcl-PHAs synthesis.

Depending on the type of carbon sources used, there are several metabolic routes that could be involved in the synthesis of 3-hydroacyl-CoA thioesters. In this study, *Pseudomonas putida* KT2440 was cultured on sodium gluconate, thus fatty acid de novo synthesis was active during the growth. The abundance of acetyl-CoA carboxylase proteins that catalyze the first step of fatty acid biosynthesis was decreased. The expression of acetyl-CoA carboxylase subunit A (spot 517) was down-regulated by 4.6-fold and 4.9-fold at 24 h and 48 h, respectively. Also, the down-regulation of acetyl-CoA carboxylase biotin carboxylase subunit (spot 513) was observed. However, these proteins levels were still high in the mcl-PHAs production phase. The production of ACC proteins was repressed when the bacteria entered the stationary phase. These data are consistent with our previous RNA-seq analysis that the transcripts levels of genes coding for acetyl-CoA carboxylase were depended on the growth phase of *P. putida* KT2440 and their overexpression may influence on the rate of polyhydroxyalkanoates biosynthesis [13].

Three ATP synthesis proteins were also differentially expressed. All of these proteins belong to FOFI-ATP synthase which is responsible for producing ATP and maintain cellular processes in bacterial cells. In the current study, the abundance of FOF1 ATP synthase subunit alfa (spot 605), FOF1 ATP synthase subunit beta (spot 495) and ATP synthase F1 beta subunit (spot 489) was increased by more than fourfold during mcl-PHAs synthesis. More abundant proteins of energy metabolism may be necessary for the accumulation of biopolymers. The increased expression of proteins related to ATP synthase was preferable to provide more energy for the polymerization of mcl-PHAs.

### Nitrogen metabolism and protein synthesis

In mcl-PHA-synthesizing *Pseudomonas putida*, proteins involved in nitrogen metabolism (nitrogen regulatory protein P-II, spot 1025 and 1026; glutamine synthetase type I, spot 573) were up-regulated relative to the control time-point of the cultivation. Nitrogen regulatory protein P-II is sensitive to nitrogen concentrations and was identified as “the central units” of nitrogen-assimilatory processes [14]. It has been proposed that P-II proteins regulate the function of the two component regulatory NtrB/NtrC system. They also control ATP-dependent assimilation of ammonia through glutamine synthetase that is a crucial enzyme in the network of nitrogen metabolism [15]. In the current study, due to nitrogen limitation, the induction of these proteins helped to increase a pool of nitrogen from alternative sources. In contrast, the abundance of glutamate dehydrogenase (spot 566) decreased dramatically by more than eightfold at both time-points. Haves et al. [16] showed that GdhA is directly repressed by NtrC. The authors proved that *gdhA* gene encoding glutamate dehydrogenase in *Pseudomonas putida* is induced by excess nitrogen. Based on our results, due to nitrogen depletion the increase in the level of nitrogen regulatory protein P-II repressed the abundance of glutamate dehydrogenase.

Moreover, proteins involved in ribosome synthesis annotated as ribosomal protein S1 (spot 506), 30S ribosomal protein S1 (spot 405) were significantly up-regulated. The protein S1 is required for the efficient in vivo translation of natural mRNAs in *Escherichia coli* cells [17]. The results suggested that they may help *P. putida* KT2440 to survive under imbalance growth conditions. However, our data showed that the expression of 50S ribosomal protein L7/L12 decreased by 2.6-fold and only at 48 h. Early studies revealed that L7/L12 was crucial for optimal translation rates, accuracy and termination [18]. It remains to be unclear why certain ribosomal proteins are overexpressed while others are underexpressed. We can speculate that this may be associated with functions that are independent of their roles in the ribosomes [19].

### Amino acid synthesis

An increased abundance of proteins involved in arginine biosynthesis: N-acetyl-gamma-glutamyl-phosphate reductase (spot 657) and arginine deiminase spot 585, were observed at 24 h and 48 h of the bioprocess, respectively. In contrast, the abundance of acetylglutamate kinase (spot 756) decreased at 24 h relative to 8 h. Due to the decrease in abundance of proteins belonging to glycolysis and TCA cycle, *Pseudomonas putida* used arginine deiminase pathway as an alternative source of energy production. Similar results were obtained in *Pseudomonas putida* KT2440 upon exposure to vanillin [20]. It was reported that the conversion of arginine to ornithine, ammonia and CO_2_ generate 1 mol of ATP per mol of arginine consumed. Probably, the analyzed mcl-PHAs producer activated the arginine biosynthesis pathway under poor growth conditions. Interestingly other differentially expressed proteins involved in amino acid metabolism were down-regulated. The expression of 3-isopropylmalate dehydrogenase (spot 580), isopropylmalate isomerase large subunit (spot 456 and 458) and keto-acid reductoisomerase (spot 708) participating in the synthesis of valine, leucine and isoleucine decreased by about 3.0-fold at the both time-points relative to 8 h. Moreover, the abundance of proteins involved in cysteine and methionine, adenosylhomocysteinase (spot 477) and S-adenosylmethionine synthetase (spot 550) was down-regulated at 24 and 48 h compared to the beginning of the bioprocess. Also, the synthesis of proteins responsible for serine metabolism (D-3-phosphoglycerate dehydrogenase, spot 565 and serine hydroxymethyltransferase, spot 557) were repressed at the same time-points. This could happen due to the induction of glutamine synthetase (spot 573) during the cultivation. Previous reports indicated that GlnA could inhibit the synthesis of proteins involved in amino acid synthesis [6]. In addition, decreased expression of these proteins could indicate that was suppressed due to nitrogen starvation [21].

### Transporters

In the genome of *Pseudomonas putida* KT2440 the genes coding for membrane transport systems constitute approximately 12% (about 370 cytoplasmic membrane transport systems) [22]. Some data showed that bacterial cells adapt the transport enzyme density in the membrane according to environmental conditions [23]. Our results indicate that ABC transporters involved in the amino acid transport (spot 680, 675, 589, 966, 619), basic amino acid specific porin OprD (spot 569) and polyamine transport (spot 721), were up-regulated at the analyzed time-points. These data were in agreement with the results obtained by Nikodinovic-Runic et al. [8]. The authors observed higher leucine and valine uptake in the samples taken from the culture of *P. putida* CA-3 grown on styrene during PHAs synthesis under nitrogen starvation.

Furthermore, the dramatically increased levels of polyamide transport protein (spot 721) by more than sevenfold could be related with stressful growth conditions. Polyamines are a group of small polycations that could play different roles in the bacterial cells such as survival, growth, gene expression, stress response and cell division [24]. Thus, polyamide cellular transport systems are the major routes to regulate polyamine levels in the cells that should be controlled to enable the efficiency of certain cellular functions [25].

Moreover, during biofermentation process *P. putida* induced proteins belong to TonB-dependent transporters (TBDTs), the bacterial outer membrane proteins that bind and transport ferric chelates called siderophores, as well as vitamin B12. TonB-dependent proteins are essential to transduce the energy across the outer membrane and into the periplasmic space prior to inner membrane translocation [26]. It was speculated that the genome of

*Pseudomonas putida KT2440* carry up to 31 putative TonB-dependent receptor genes [27]. In our experiment, the significant abundance in two TonB-dependent receptor (spot 210, 189) and outer membrane ferric siderophore receptor (spot 737) was observed. The induction of these proteins was caused by the involvement of bacterial cell in the uptake of iron and vitamin B12 for the bacterial growth during the conducted bioprocess. Other identified proteins involved in transport were also up-regulated suggesting that they all serve as a signal for response to the low concentration of nutrients essential for the metabolism of *P. putida* KT2440 cells.

### Stress response

Under stress conditions during bacterial growth, protein folding could be affected. The ability of microorganisms to sense and respond to alterations in their environment is essential for their survival. PHAs are synthesized in bacterial cells as insoluble granules that form primarily a reservoir of carbon and energy. Recent research revealed protective features of PHAs in the response of a wide range of stress factors, thus conferring their general fitness and robustness [28].

In the current study, the expression of universal stress protein (spot 958 and 993) increased by 2.2-fold and 2.4-fold at 24 h and 48 h relative to control, respectively. The abundance of proteins such as OsmC family protein (spot 991) and catalase/peroxidase HPI protein (spot 331) involved in detoxification of reactive oxygen species (ROS) was also upregulated after 8 h of the bioprocess. *Pseudomonas putida* is an obligate aerobe generating energy via carbon source oxidation which needs oxygen to form water. Our results indicate that the production of ROS increased and there was a need to repair proteins damaged by ROS. Moreover, the imbalance in carbon and nitrogen assimilation could lead to oxidative stress. The same observations were made during lipid biosynthesis under imbalanced conditions [29].

In contrast, chaperones (DnaK, spot 365 and GroEL, spot 455 and 735) involved in the recovery of protein aggregates, heat shock protein 90 (spot 348 and 346) involved in protein folding and stabilization and polynucleotide phopshorylase/polyadenylase (PNPase, spot 353), a critical enzyme in RNA metabolism, were significantly down-regulated at 24 h and 48 h besides nitrogen depletion. The highest expression of these proteins was observed at the beginning of the bioprocess due to the accumulated mcl-PHAs granules that could be stressors for the cells. So far, in metabolically engineered *E. coli* strains harboring heterologous PHAs biosynthesis genes, the heat shock proteins expression was induced by the accumulation of the biopolyester inclusions [30]. PNPase serves as a component of an RNA degradation machine [31]. Also, DnaK and GroEL are associated with degradosomes. PNPase together with Rnase E have been proposed to take part in the degradation of specific set of mRNAs coding for proteins involved in macromolecule biosynthesis and modification. Moreover, the previous evidence showed that PNPase could have an effect on genes involved in glycolytic and cysteine biosynthesis pathways [32]. In our study, the abundance of proteins involved in this biosynthetic pathways were also decreased at 24 h and 48 h compared to the beginning of the cultivation.

The mcl-PHAs accumulation in bacterial cells alters the protein abundance involved in general stress response and maintenance of cellular homeostasis. Membrane proteins play an essential role in various cellular responses and in the bacterial adaptation to changes of environmental conditions [33]. Outer membrane proteins are the major components of Gram-negative bacterial outer membrane. These proteins primarily function in flagellum assembly, pore formation, transport of specific substrates, and outer membrane stabilization [34]. We identified flagellin FliC (spot 388) and OmpF family protein (spot 590, 609, 645) as differentially expressed outer membrane proteins. The abundance of FliC protein significantly decreased by 7.9-fold and 9.9-fold at 24 h and 48 h relative to the control, respectively. In contrast, at the same time-points, proteins involved in maintaining several degrees of permeability to the bacterial cells (OmpF) was dramatically up-regulated by more than 17-fold and 15-fold. The up-regulation of YVTN family beta-propeller repeat-containing protein was also observed but only at the end of the biofermentation. The function of this membrane protein remain to be unknown.

The high expression level of protein chain elongation factors EF-TuA (spot 671), EF-TuB (spot 540, 776) and EF-Ts (spot 837 and 717) was observed during the mcl-PHAs synthesis bioprocess. Elongation factors thermo unstable (EF-Tu) are one of the most abundant proteins in bacteria that acts as a carrier of amino acyl-tRNA to the ribosome during protein synthesis [35]. Previous reports indicated that EF-Tu in *Bacillus subtilis* plays a second role in cell shape maintenance [36]. Our results suggested that both EF-Tu are induced at 24 h and 48 h while the *P. putida* KT2440 shape was disturbed due to intracellularly accumulated mcl-PHAs granules. Moreover, EF-Ts play a role in the contribution of rapid and faithful protein synthesis [37].

Furthermore, the abundance of cold-shock protein (spot 1022) and thiazole synthase (spot 731) increased at 24 and 48 h (stationary growth phase) of the mcl-PHAs biosynthesis process, respectively. In *E. coli*, cold-shock proteins have been linked to the inhibition of DNA replication and their induction was observed during stationary phase to resign growth [38]. Cold-shock responses have been also proposed to aid misfolded RNA to adopt functional conformation and thus suppress harmful mutations that affect RNA structure [39]. Moreover, the up-regulation of thiazole biosynthetic enzyme (spot 731) associated with thiamine metabolism could be associated with the cellular response to completely nitrogen depletion and DNA damage tolerance [21].

### Other

Mcl-PHA-accumulated cells showed a significant expression of protein involved in cell chemotaxis (chemotaxis protein CheX, spot 676). This protein was dramatically down-regulated at 24 h and 48 h suggested the repression of the cell motility in response to stress. Motility is an energy-consuming function, so the downregulation of the protein coding for motility may serve as an adaptive response of *P. putida* KT2440 to save energy in coping with unfavorable conditions during cell growth [40]. Also, the abundance of hypothetical protein (PP_3089, spot 862) linked with biofilm formation process decreased in response to environmental stressors in the early stationary phase (24 h).

Moreover, the expression of adenylosuccinate lyase (spot 553), nucleoside-diphosphate kinase (spot 918) and bifunctional phosphoribosyl aminoimidazole carboxamide formyltransferase/IMP cyclohydrolase (spot 448) associated with nucleic acid metabolism decreased at 24 and 48 h of the bioprocess. It indicates that the bacterial cell reproduction started to slow down. As can be seen on Fig. 1 *Pseudomonas putida* KT2440 reached the stationary phase at these time-points.

## Conclusions

This study presents a proteomic analysis of mcl-PHAs synthesis by *Pseudomonas putida* KT440 grown on sodium gluconate under nitrogen deficiency conditions. The data proved that nitrogen deprivation stimulated the biopolyesters production. The physiological response of *P. putida* KT2440 to environmental stimuli during mcl-PHAs synthesis has a multifaceted nature. The presented data allow us to investigate time-course proteome rearrangement in response to nitrogen limitation and biopolyesters accumulation. We expected a significantly increased abundance of proteins involved in PHAs synthesis and accumulation process. However, PhaC1, PhaZ, PhaC2, PhaD, PhaF and PhaI have not been identified in the 2D-DIGE experiment as differentially abundant proteins. It suggests that a concentration of these proteins was too low to be detected. However, our proteomic analysis confirmed that several other proteins have significantly changed their abundance in response to mcl-PHAs synthesis under nitrogen starvation.

The abundance of proteins involved in carbon metabolism were significantly decreased at 24 h and 48 h of the cultivations. The suppression of glycolytic and TCA pathway lead more carbon flux to acetyl-CoA synthesis, and thus increasing the mcl-PHAs in *P. putida* KT2440 cells. The enzymes involved in the above-mentioned pathways have an essential role in balancing reducing equivalent. It is well known that elevated intracellular ratios of NADH/NAD^+^ and NADPH/NADP^+^ are important for biopolyesters synthesis [7]. In addition, due to their down-regulation, the analyzed strain used arginine deiminase pathway as a source of energy production. Furthermore, the up-regulation of TonB-dependent proteins was associated with the uptake of iron and vitamin B12 that are essential for the growth. The expression of proteins involved in nucleic acid metabolism decreased suggesting that this shifts the metabolism of *P. putida* to mcl-PHAs biosynthesis. Moreover, our results indicate that mcl-PHAs accumulated in the bacterial cells changed the protein abundance involved in stress response and cellular homeostasis. The results indicate that the above mentioned proteins could be the appropriate candidates for improving biopolymers content in bacterial cells and for monitoring a bacterial response to environmental factors.

## Methods

### Fermentation condition

*Pseudomonas putida* KT2440 (ATCC^®^ 47054™) from long-term storage were firstly grown overnight in lysogeny broth (1% w/v tryptone, 0.5% w/v yeast extract, 1% NaCl) at 30 °C at 220 rpm in a rotary shaker. Then, the bacterial cells were transferred to a mineral medium for PHAs synthesis. The PHAs production medium consisted of (per liter): 3.5 g Na_2_HPO_4_·12H_2_O, 7.0 g KH_2_PO_4_, 1 g (NH_4_)SO_4_, 1 g MgSO_4_·7H_2_O, 10 g sodium gluconate and 2.5 mL of trace element solution. Each liter of trace element solution contained the following components: 20 g FeCl_3_·6H_2_O, 10 g CaCl_2_·H_2_O, 0.03 g CuSO_4_·5H_2_O, 0.05 g MnCl_2_·4H_2_O, 0.1 g ZnSO_4_·7H_2_O dissolved in 0.5 N HCl. The cultivation was carried out in a 7 L bioreactor (Biostat A, Sartorius, Germany) at 30 °C with an aeration rate of 4 L/min. pH-value was maintained at 7 through the modulated addition of concentrated 1 N NaOH and 1 N HCl. The dissolved oxygen was monitored during the whole cycle with O_2_ electrode (InPro 6800, Mettler Toledo GmbH, Switzerland) and maintained 50% air saturation by adjusting the agitation rate from 300 rpm to 1000 rpm automatically. Total fermentation time was 48 h. Three replicate cultures were conducted.

### Analytical procedures

The cell density of the *Pseudomonas putida* KT2440 was monitored by measuring the absorbance at 600 nm (OD_600_) using a spectrophotometer. During the cultivations, culture samples were taken for analysis at 8, 17, 24, 32, 41 and 48 h for measurements of cell dry weight, mcl-PHAs accumulation, ammonium concentration and for determination of monomers composition and their concentrations. Cell dry weight (CDW) was determined by centrifuging 100 ml culture broth at 11.200×*g* for 10 min. The collected cells were then weighed after lyophilization. The lyophilization process was performed by Lyovac GT2 System (SRK Systemtechnik GmbH) for 24 h. Ammonium concentration was measured spectrophotometrically using the Hach Lange DR 2800 spectrophotometer (Hach Lange, Düsseldorf DE) and the LCK303 kit according to the manufacturer’s instructions.

Mcl-PHAs were extracted from lyophilized cells using the chloroform/methanol procedure for quantitative and qualitative analysis of biopolymers. The monomeric composition of the purified mcl-PHAs was determined using a methanolysis protocol as described previously [41]. The concentrations of methyl esters were estimated by a gas chromatography (GC) equipped with a capillary column Varian VF-5 ms with a film thickness of 0.25 μm (Varian, Lake Forest, USA). Pure standards of methyl 3-hydroxy-hexanoate, -octanoate, -nonanoate, -decanoate, -undecanoate, -dodecanoate, -tetradecanoate, -hexadecanoate were purchased from Larodan Fine Chemicals (Sweden) to generate calibration curves for the methanolysis assay. All samples were analyzed in triplicates.

### Preparation of *Pseudomonas putida* KT2440 cells for proteomic analysis

Prior to proteomic analysis, the fresh bacterial cells were harvested from the cultures at 8 h (a trace amount of mcl-PHAs), 24 h (fast mcl-PHAs accumulation) and 48 h (slow mcl-PHAs accumulation) and centrifuged at 10,000×*g* at 4 °C for 5 min. Protein extracts were purified using a Clean-Up Kit (GE Healthcare, Uppsala, Sweden) according to the manufacturer’s protocol. The resulting pellets were resuspended in 100 μL of DIGE Labelling Buffer consisting of 7 M urea, 2 M thiourea, 4% w/v CHAPS and 30 mM Tris to a protein concentration of 5–10 mg/mL. The protein concentration was measured by a Coomassie (Bradford) Assay Kit (ThermoScientific, Rockford, USA) with bovine serum albumin as the standard. Proteins from three biological replicates of each time point of the cultivations were used for the proteomic study.

### Fluorescent labelling with CyDyes and 2D-DIGE analysis

Aliquot of 50 µg of protein from each sample (n = 3 for each time point) was dissolved in a labelling buffer (7 M urea, 2 M thiourea, 4% w/v CHAPS, 30 mM Tris, pH 8.0). Protein fractions were labelled with CyDye DIGE Fluor minimal dyes (GE Healthcare, Uppsala, Sweden) reconstituted in 99.8% anhydrous DMF 400 pmol of either Cy3 or Cy5 fluorescent dyes (GE Healthcare, IL, USA) for comparison on the same gel. An internal standard containing a pool of all samples was labelled with Cy2 fluorescent dye, and this was used as a standard on all gels to aid image matching and cross-gel statistical analysis. Labelling reactions were performed on ice in the dark for 30 min. Samples labeled with Cy3 (50 µg) were mixed with samples labeled with Cy5 (50 µg) and 50 µg of Cy2-labeled internal standard, and then rehydration solution was added (7 M urea, 2 M thiourea, 2% CHAPS, DDT, 2% pharmalyte pH 3–10, and 130 mM DTT) to a final volume of 450 µL. The labelled samples were loaded on 24-cm Immobiline DryStrips, at nonlinear pH range 3–10 (GE Healthcare), and rehydrated for 12 h. Isoelectric focusing (IEF) was run using an Ettan IPGphor apparatus (GE Healthcare) at 20 °C with current limited to 50µA per strip and the following voltage program: 500 V/2 h, a linear gradient to 1000 V over 1 h and a linear gradient to 10,000 V over 3 h, then an 10,000 V constant for 4 h. After that, the IPG strips were equilibrated in equilibration buffer (6 M urea, 75 mM Tris–HCl, pH 8.8, 29.3% glycerol, 2% sodium dodecyl sulfate, 0.002% bromophenol blue) containing 65 mM DDT for 15 min, and then containing 135 mM iodoacetamide for 15 min. As a second dimension, 12.5% precast DIGE gels Ettan DALT (gel size 25.5 × 19.6 cm, 1 mm thickness, GE Healthcare) were run at 1.5 W/gel for 16 h in the Ettan Dalt-Six apparatus (GE Healthcare).

### Image analysis

After 2D-DIGE electrophoresis, the CyDye-labelled gels were scanned with a Typhoon 9500 FLA scanner (GE Healthcare) using the parameters recommended by the manufacturer. The SameSpots software (Totallab, Newcastle, UK) was used to match and analyze protein spots. Gels were aligned automatically and the alignment was refined manually. Differential in-gel analysis was used to calculate protein abundance alterations between samples on the same gel. The resulting spot maps for each biological replicate were then analyzed through biological variation analysis to provide statistical data on the differential protein expression. Spots that exhibited a change of the cumulated normalized abundance from all replicates of at least 2.0 and a *p* value < 0.05 were considered as differentially regulated.

### Spot picking, protein digestion and MALDI-TOF/TOF protein identification

DIGE gels were restained using Coomassie Brilliant Blue G-250 (Bio-Rad, Hercules, CA) in order to properly pick the differentially expressed proteins. Spots presenting significant differences were excised from gels and digested overnight using modified sequencing grade trypsin (Promega, Madison, USA). After digestion, the spots were concentrated and desalted using Zip-Tip C18 tips (Millipore, Billerica, USA). Each Zip-Tip was first washed with 100% acetonitrile (ACN), then equilibrated with 50% ACN in 0.1% TFA and 0.1% TFA in water. Then, the peptides were loaded onto the Zip-tip and eluted with 2 μL of 50% ACN in 0.1% TFA. The eluted sample was mixed with 2 μL of the matrix solution (5 mg α-cyano-4-hydroxycinnamic acid (Bruker Daltonics, Billerica, USA) in 1 mL of 50% ACN in 0.1% TFA). The mixture was spotted onto the MALDI target plate (MT 34 Target Plate Ground Steel (Bruker Daltonics, Billerica, USA) and left to dry. MALDI-TOF/TOF MS analysis was carried out using a time-of-flight Autoflex-ToF/ToF mass spectrometer (MALDI-TOF/TOF, Bruker Daltonics, Billerica, USA). The collected MS and MS/MS LIFT spectra of selected ions were externally calibrated using monoisotpoic [M+H] + ion peptide calibration standards (Bruker Daltonics). Collected spectra were imported to BioTools (Bruker Daltonics, Billerica, USA) and searched using BioTools as a front end. MS peptide mass fingerprint (PMF) and fragment mass spectra (MS/MS) from each individual spot were combined and searched on an in-house Mascot Server (Matrix Science, London, UK). Trypsin was specified as cleavage enzyme allowing two missed cleavages. Alkylation of cysteine by carbamidomethylation was set as a fixed modification and oxidation of methionine was applied as a variable modification. Mass tolerance mono was set to 50 ppm, fragment ions to 0.5 Da and parent ion to 200 ppm. For the PMF and MS/MS ion search, statistically significant (p ≤ 0.05) matches with at least two correctly identified parent ions by MASCOT were regarded as correct hits.

### Gene ontology annotation and bioinformatic analysis

The analyses were conducted with the differentially expressed proteins. Gene Ontology Annotation (GO) was determined by matching GI numbers of identified proteins to the UniProtKB database (http://www.uniprot.org) to obtain GO annotation. The Kyoto Encyclopedia of Genes and Genomes (KEGG) was used for an annotation of biological pathways involved in mcl-PHAs biosynthesis process. Furthermore, the analysis of potential protein–protein interactions were performed with STRING software, version 10.5 (https://string-db.org) that enables to create interactions for known or hypothetical proteins, including associations with genomic contexts, high-throughput experiments and co-expression. Principal component analysis (PCA) and hierarchical clustering of significantly and differentially expressed proteins was performed using a web-based ClustalVis software (https://biit.cs.ut.ee/clustvis) to group samples with similar expression patterns into clusters. PCA was performed to visualize differences between samples. PCA creates and coordinates those points using the total log standardized abundance of spots on a certain gel. That allow to summarize the variation in the data from protein spots in the form of principal components, where the first component explains the largest proportion of the variance.

### Statistical analysis

The data represents the mean ± standard deviation (SD) of three independent experiments. The dependence between the biomass concentration and mcl-PHAs content in *P. putida* KT2440 cells was calculated using the Spearman’s rank correlation coefficient (Statistica, Statsoft Inc., USA). Statistical analysis of the changes in protein abundance was performed using the SameSpots software in three biological replicates (individual cultivations). Experimental groups were compared using a Student’s t-test. Changes in protein spot abundance were considered statistically significant at p < 0.05, with a fold change of ± 2.0. Normalised spot intensities on gels were compared a Student’s t-test at a significance level of 0.05.

## Additional files

**Additional file 1: Table S1.** Identification of differentially expressed proteins at 24 h and 48 relative to 8 h of the *Pseudomonas putida* KT2440 fermentation during mcl-PHAs synthesis.

**Additional file 2: Figure S1.** Search Tool for the retrieval of Interacting Genes/Proteins (STRING) analysis of identified proteins (A) at 24 h and (B) 48 h compared to 8 h of the cultivation. Full proteins name can be found in the UniProtKB database.

## Data Availability

Not applicable.
