# Supplementary material for: A 2D-DIGE-based proteomic analysis brings new insights into cellular responses of Pseudomonas putida KT2440 during polyhydroxyalkanoates synthesis
Source: Microb Cell Fact. 2019 May 28;18:93. doi: 10.1186/s12934-019-1146-5 (PMC6537436; doi:10.1186/s12934-019-1146-5)
Supplement: Supplementary file 1 — Additional file 1: Table S1. Identification of differentially expressed proteins at 24 h and 48 relative to 8 h of the Pseudomonas putida KT2440 fermentation during mcl-PHAs synthesis. [file 12934_2019_1146_MOESM1_ESM.docx]

| Spot no.  Table S1. Identification of differentially expressed proteins at 24 h and 48 relative to 8 h of the *Pseudomonas putida* KT2440 fermentation during   mcl-PHAs synthesis | Identified protein | Accession number | p-value | Fold change 24 h_8 h | Fold change 48 h_8 h | pI | MW (Da) | Protein score | No. of peptides (ion score >30) | Sequence coverage (%) |
| --- | --- | --- | --- | --- | --- | --- | --- | --- | --- | --- |
| 474 | dihydrolipoamide dehydrogenase | gi\|26990879 | 9.22E-04 | - 2.4 | nd | 5.93 | 50109 | 170 | 3 | 22 |
| 478 | dihydrolipoamide dehydrogenase | gi\|26990879 | 1.56E-04 | - 5.2 | - 4.2 | 5.93 | 50109 | 156 | 2 | 16 |
| 557 | serine hydroxymethyltransferase | gi\|26987407 | 6.32E-05 | - 6.1 | - 4.6 | 5.85 | 45154 | 203 | 2 | 36 |
| 730 | succinate--CoA ligase subunit alpha | gi\|969975279 | 3.22E-05 | - 3.4 | - 2.8 | 5.78 | 30526 | 282 | 3 | 70 |
| 603 | succinyl-CoA ligase subunit beta | gi\|489347078 | 0.001 | - 3.3 | - 2.5 | 5.83 | 41499 | 188 | 3 | 44 |
| 614 | succinyl-CoA synthetase subunit beta | gi\|26990878 | 1.758e-008 | nd | - 5.2 | 5.83 | 41499 | 433 | 4 | 49 |
| 517 | acetyl-CoA carboxylase subunitA | gi\|26992023 | 3.44E-04 | - 4.6 | - 4.9 | 6.07 | 52309 | 101 | 2 | 13 |
| 464 | hydro-lyase. Fe-S type. tartrate/fumarate subfamily. alpha subunit | gi\|26987633 | 3.54E-05 | - 3.1 | - 3.5 | 5.14 | 55017 | 277 | 4 | 13 |
| 684 | type I glyceraldehyde-3-phosphate dehydrogenase | gi\|26987745 | 7.04E-04 | - 2.9 | - 2.7 | 6.49 | 36320 | 234 | 2 | 48 |
| 625 | fructose-1.6-bisphosphate aldolase | gi\|26991638 | 1.55E-04 | - 3.2 | - 2.5 | 5.48 | 38713 | 383 | 3 | 45 |
| 925 | carbohydrate kinase. thermoresistant glucokinase family | gi\|26990129 | 5.53E-05 | - 5.5 | nd | 5.58 | 19228 | 152 | 2 | 40 |
| 510 | acetyl-CoA carboxylase biotin carboxylase subunit | gi\|26987296 | 0.002 | - 2.4 | nd | 6.09 | 49456 | 167 | 2 | 19 |
| 513 | acetyl-CoA carboxylase biotin carboxylase subunit | gi\|26987296 | 2.08E-04 | - 3.2 | - 2.8 | 6.09 | 49456 | 81 | 2 | 8 |
| 752 | 6-phosphogluconolactonase | gi\|26987759 | 6.54E-04 | - 2.7 | - 2.5 | 6.23 | 25631 | 343 | 4 | 58 |
| 408 | phosphogluconate dehydratase | gi\|26987746 | 0.001 | - 2.3 | -2.2 | 6.00 | 65774 | 233 | 4 | 18 |
| 440 | succinate dehydrogenase flavoprotein subunit | gi\|26990883 | 0.003 | - 2.3 | nd | 5.83 | 63979 | 509 | 4 | 30 |
| 748 | 5.10-methylenetetrahydrofolate reductase | gi\|26991654 | 2.57E-05 | - 2.7 | Nd | 5.86 | 33187 | 249 | 2 | 56 |
| 632 | beta-ketothiolase | gi\|26990459 | 6.185e-004 | nd | - 2.1 | 6.41 | 41133 | 140 | 2 | 30 |
| 605 | F0F1 ATP synthase subunit alpha | gi\|26992090 | 1.25E-05 | 9.5 | 9.4 | 5.38 | 55489 | 320 | 4 | 23 |
| 495 | F0F1 ATP synthase subunit beta | gi\|26992088 | 0.043 | 4.4 | 4.0 | 4.88 | 49385 | 94 | 2 | 23 |
| 489 | ATP synthase F1. beta subunit | gi\|254520882 | 5.13E-05 | 6.2 | 5.8 | 5.09 | 50899 | 193 | 2 | 35 |
| 573 | glutamine synthetase. type I | gi\|26991722 | 0.001 | 5.1 | 4.7 | 5.21 | 51936 | 188 | 2 | 12 |
| 1025 | nitrogen regulatory protein P-II (Activator of NRII(GlnL/NtrB) phosphatase) | gi\|26991910 | 0.001 | 4.8 | 3.1 | 5.42 | 12323 | 136 | 2 | 53 |
| 1026 | nitrogen regulatory protein P-II (Activator of NRII(GlnL/NtrB) phosphatase) | gi\|26991910 | 0.038 | 2.4 | nd | 5.42 | 12323 | 115 | 2 | 42 |
| 566 | glutamate dehydrogenase | gi\|26987411 | 3.71E-05 | - 9.0 | - 8.4 | 6.12 | 49296 | 178 | 2 | 29 |
| 787 | pyrroline-5-carboxylate reductase | gi\|26990483 | 2.53E-04 | - 2.3 | - 2.1 | 5.84 | 27868 | 221 | 3 | 34 |
| 607 | dTDP-glucose 4.6-dehydratase | gi\|26988516 | 6.41E-04 | 2.1 | nd | 5.76 | 40949 | 174 | 2 | 41 |
| 365 | chaperone protein DnaK | gi\|26991410 | 2.08E-05 | - 4.3 | - 3.6 | 4.83 | 68873 | 409 | 4 | 17 |
| 348 | heat shock protein 90 | gi\|26990871 | 2.57E-05 | - 5.0 | - 5.0 | 5.21 | 71567 | 349 | 4 | 24 |
| 346 | heat shock protein 90 | gi\|26990871 | 0.002 | - 3.8 | - 4.3 | 5.21 | 71567 | 85 | 3 | 14 |
| 455 | chaperonin GroEL | gi\|26988095 | 1.19E-06 | - 3.4 | - 3.0 | 4.97 | 56765 | 439 | 4 | 32 |
| 735 | chaperonin GroEL | gi\|26988095 | 1.06E-05 | 3.7 | 3.9 | 4.97 | 56765 | 162 | 2 | 7 |
| 331 | catalase/peroxidase HPI | gi\|26990379 | 0.012 | 2.6 | 2.3 | 5.64 | 82068 | 323 | 4 | 17 |
| 991 | OsmC family protein | gi\|26988589 | 2.09E-05 | 3.3 | 3.8 | 6.28 | 14770 | 104 | 2 | 23 |
| 958 | universal stress protein | gi\|26988856 | 0.001 | 2.2 | nd | 6.14 | 16369 | 125 | 2 | 39 |
| 993 | universal stress protein | gi\|26988856 | 0.016 | nd | 2.4 | 6.14 | 16369 | 125 | 2 | 39 |
| 1022 | cold-shock domain-contain protein | gi\|26987721 | 0.002 | 2.3 | Nd | 9.98 | 18860 | 183 | 2 | 42 |
| 766 | two-component system response regulator /DNA binding response regulator | gi\|26989071 | 0.01 | 2.6 | 3.6 | 5.09 | 24933 | 137 | 2 | 24 |
| 731 | thiazole synthase | gi\|26991780 | 0.012 | nd | 2.0 | 5.74 | 29184 | 123 | 3 | 42 |
| 565 | D-3-phosphoglycerate dehydrogenase | gi\|26991831 | 1.18E-04 | - 4.8 | - 4.2 | 5.93 | 44539 | 211 | 4 | 33 |
| 580 | 3-isopropylmalate dehydrogenase | gi\|26988714 | 2.69E-05 | - 3.5 | - 3.4 | 5.03 | 38971 | 137 | 2 | 29 |
| 456 | isopropylmalate isomerase large subunit | gi\|26988711 | 8.89E-04 | - 3.3 | - 2.7 | 5.55 | 51679 | 200 | 3 | 19 |
| 458 | isopropylmalate isomerase large subunit | gi\|26988711 | 1.64E-04 | - 2.8 | - 2.6 | 5.55 | 51679 | 139 | 2 | 16 |
| 708 | ketol-acid reductoisomerase | gi\|26991362 | 1.22E-04 | - 2.7 | - 2.6 | 5.48 | 36575 | 416 | 4 | 23 |
| 550 | S-adenosylmethionine synthetase | gi\|26991645 | 0.001 | - 5.2 | - 4.7 | 5.27 | 43152 | 163 | 2 | 40 |
| 477 | adenosylhomocysteinase | gi\|1002826361 | 1.11E-05 | - 3.2 | - 3.0 | 5.42 | 51870 | 381 | 5 | 30 |
| 470 | adenosylhomocysteinase | gi\|1002826361 | 4.16E-06 | - 2.3 | Nd | 5.42 | 51870 | 198 | 2 | 24 |
| 756 | acetylglutamate kinase | gi\|26991965 | 7.80E-05 | - 2.4 | Nd | 5.57 | 31990 | 247 | 2 | 26 |
| 657 | N-acetyl-gamma-glutamyl-phosphate reductase | gi\|26987173 | 2.90E-06 | 9.7 | Nd | 6.30 | 36540 | 80 | 2 | 24 |
| 585 | arginine deiminase | gi\|26987737 | 0.018 | nd | 2.0 | 5.57 | 46775 | 268 | 4 | 18 |
| 506 | ribosomal protein S1 | gi\|24983268 | 8.98E-04 | 8.5 | 9.7 | 4.83 | 63637 | 105 | 2 | 9 |
| 671 | elongation factor Tu-A | gi\|26987181 | 0.0007959 | 3.2 | 2.8 | 5.36 | 43180 | 121 | 2 | 11 |
| 570 | elongation factor Tu-B | gi\|26987193 | 8.62E-04 | 3.4 | 3.7 | 5.36 | 43180 | 183 | 2 | 43 |
| 776 | elongation factor Tu-B | gi\|26987193 | 0.003 | 7.0 | 5.5 | 5.22 | 43793 | 84 | 2 | 31 |
| 837 | elongation factor Ts | gi\|26988324 | 0.003 | 4.2 | 4.0 | 5.14 | 30527 | 85 | 2 | 14 |
| 405 | 30S ribosomal protein S1 | gi\|1002825963 | 0.002 | 2.3 | 2.2 | 5.03 | 61568 | 342 | 4 | 17 |
| 298 | elongation factor G | gi\|26987192 | 0.014 | - 2.4 | nd | 5.13 | 79053 | 233 | 2 | 22 |
| 505 | aspartyl/glutamyl-tRNA amidotransferase subunit B | gi\|26987666 | 0.04 | - 2.1 | nd | 5.06 | 52930 | 182 | 2 | 15 |
| 909 | 50S ribosomal protein L7/L12 | gi\|26987187 | 3.135e-004 | nd | - 2.6 | 4.74 | 12592 | 178 | 2 | 19 |
| 828 | YVTN family beta-propeller repeat-containing protein | gi\|26992068 | 0.015 | nd | 2.1 | 6.75 | 39387 | 80 | 2 | 24 |
| 680 | general amino acid ABC transporter periplasmic binding protein | gi\|26988032 | 7.75E-04 | 5.2 | 4.4 | 5.84 | 36630 | 350 | 5 | 49 |
| 675 | general amino acid ABC transporter. periplasmic binding protein | gi\|26988032 | 6.05E-05 | 3.8 | 3.8 | 5.84 | 36630 | 98 | 2 | 30 |
| 589 | branched chain amino acid ABC transporter substrate-binding protein | gi\|653579077 | 0.003 | 2.3 | 2.3 | 5.75 | 40518 | 290 | 3 | 37 |
| 661 | extracellular ligand-binding receptor | gi\|26991547 | 0.005 | 4.2 | 5.9 | 5.93 | 40417 | 88 | 2 | 17 |
| 721 | polyamine ABC transporter. periplasmic polyamine-binding protein | gi\|26987153 | 2.33E-05 | 7.8 | 7.0 | 5.62 | 37891 | 88 | 2 | 19 |
| 590 | OmpF family protein | gi\|26988814 | 0.002 | 2.3 | 2.2 | 4.72 | 37217 | 288 | 2 | 36 |
| 645 | porin F | gi\|26988814 | 1.24E-06 | 17.7 | 15.6 | 5.03 | 39459 | 264 | 4 | 19 |
| 646 | OmpF family protein | gi\|26988814 | 0.029 | nd | 2.2 | 4.72 | 37217 | 293 | 4 | 22 |
| 609 | OmpF family protein | gi\|26988814 | 0.003 | 2.2 | nd | 4.72 | 37217 | 293 | 4 | 22 |
| 619 | ABC transporter. periplasmic binding protein | gi\|26988458 | 1.29E-04 | 2.5 | 2.6 | 5.81 | 38669 | 85 | 2 | 10 |
| 966 | amino acid ABC transporter. periplasmic amino acid-binding protein | gi\|26987807 | 0.002 | 10.5 | 9.4 | 8.61 | 33521 | 281 | 3 | 23 |
| 737 | outer membrane ferric siderophore receptor | gi\|1002825901 | 2.50E-04 | 2.3 | 2.4 | 5.36 | 31624 | 113 | 2 | 43 |
| 657 | long-chain fatty acid transporter | gi\|26988421 | 2.90E-06 | 9.7 | 7.1 | 6.67 | 49898 | 125 | 2 | 14 |
| 569 | basic amino acid specific porin OprD | gi\|26987941 | 0.006 | 2.9 | 2.2 | 4.84 | 46092 | 455 | 3 | 31 |
| 448 | bifunctional phosphoribosylaminoimidazolecarboxamide formyltransferase/IMP cyclohydrolase | gi\|26991502 | 7.17E-04 | - 3.5 | - 3.2 | 6.06 | 57945 | 128 | 2 | 12 |
| 553 | adenylosuccinate lyase | gi\|26990721 | 4.54E-04 | - 3.6 | - 3.4 | 5.69 | 50683 | 263 | 2 | 39 |
| 918 | nucleoside-diphosphate kinase | gi\|26987585 | 2.14E-05 | - 6.7 | nd | 5.45 | 15035 | 232 | 2 | 35 |
| 676 | hypothetical protein PP_3783 (chemotaxis protein CheX) | gi\|26990488 | 0.002 | - 7.8 | - 5.4 | 5.55 | 35030 | 234 | 2 | 62 |
| 640 | Serine-type D-Ala-D-Ala carboxypeptidase | gi\|26991483 | 6.61E-04 | - 2.8 | - 2.1 | 6.16 | 42375 | 225 | 3 | 43 |
| 495 | D-hydantoinase | gi\|37222601 | 0.035 | 2.7 | 2.3 | 5.71 | 52766 | 327 | 3 | 39 |
| 772 | D-hydantoinase/ dihydropyrimidinase | gi\|1001555944 | 0.026 | 2.5 | nd | 5.72 | 54790 | 205 | 2 | 17 |
| 265 | dehydrogenase subunit putative | gi\|26988393 | 8.23E-06 | 5.1 | 4.9 | 5.54 | 81426 | 342 | 5 | 18 |
| 810 | isochorismatase superfamily hydrolase | gi\|26987447 | 8.67E-06 | 11.6 | nd | 5.43 | 23126 | 188 | 2 | 27 |
| 189 | putative TonB dependent receptor | gi\|190575844 | 3.69E-04 | 3.2 | 2.3 | 5.20 | 103730 | 94 | 2 | 13 |
| 210 | B12 family TonB-dependent receptor | gi\|26987263 | 0.009 | 4.3 | 4.3 | 5.66 | 100743 | 208 | 3 | 13 |
| 934 | hypothetical protein PP_0258 | gi\|26987000 | 4.67E-05 | 4.6 | 4.1 | 5.43 | 15606 | 424 | 3 | 70 |
| 389 | hypothetical protein PP_0765 | gi\|26987501 | 9.62E-04 | 2.3 | 2.2 | 5.18 | 68123 | 201 | 2 | 33 |
| 771 | hypothetical protein PP_3787 | gi\|26990492 | 0.032 | nd | -2.4 | 6.35 | 33650 | 145 | 2 | 47 |
| 494 | PhoH family protein | gi\|26988026 | 0.0003737 | - 3.2 | - 2.7 | 5.54 | 52052 | 297 | 3 | 25 |
| 1018 | hypothetical protein PP_0998 | gi\|26987734 | 0.005 | 3.5 | 3.5 | 6.13 | 16083 | 117 | 2 | 52 |
| 353 | polynucleotide phosphorylase/polyadenylase | gi\|26991392 | 0.006 | - 2.3 | - 2.2 | 5.12 | 75192 | 278 | 5 | 11 |
| 388 | flagellin FliC | gi\|26991067 | 4.69E-05 | - 7.9 | - 9.9 | 4.39 | 67806 | 438 | 5 | 13 |
| 862 | hypothetical protein PP_3089 | gi\|26989808 | 1.21E-04 | - 3.4 | nd | 5.42 | 19555 | 348 | 2 | 43 |
