# Supplementary material for: A 2D-DIGE-based proteomic analysis brings new insights into cellular responses of Pseudomonas putida KT2440 during polyhydroxyalkanoates synthesis
Source: Microb Cell Fact. 2019 May 28;18:93. doi: 10.1186/s12934-019-1146-5 (PMC6537436; doi:10.1186/s12934-019-1146-5)
Supplement: Supplementary file 2 — Additional file 2: Figure S1. Search Tool for the retrieval of Interacting Genes/Proteins (STRING) analysis of identified proteins (A) at 24 h and (B) 48 h compared to 8 h of the cultivation. Full proteins name can be found in the UniProtKB database. [file 12934_2019_1146_MOESM2_ESM.pdf]

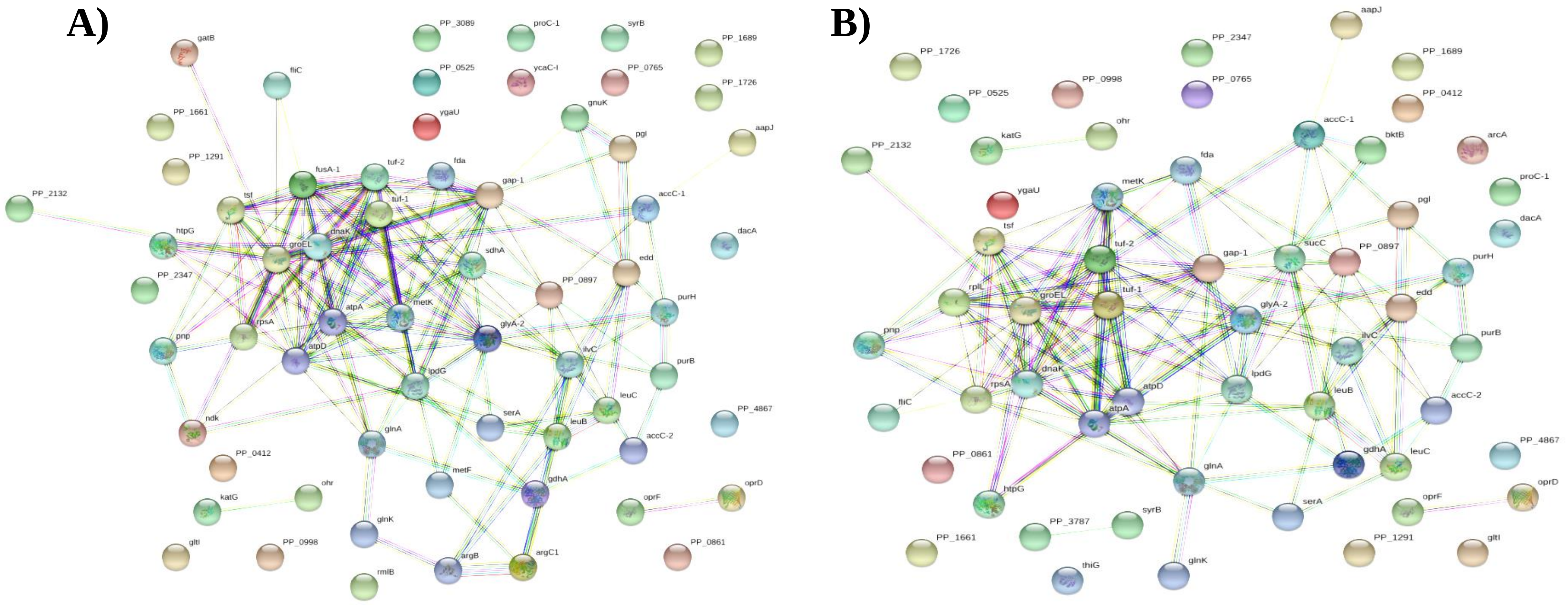
A)
gatB
fliC
PP_1661
PP_1291
PP_2132
PP_3089
proC-1
syrB
PP_1689
PP_0525
ycaC-I
PP_0765
PP_1726
ygaU
gnuK
aapJ
pgl
fusA-1
tuf-2
fda
gap-1
tsf
tuf-1
accC-1
htpG
groEL
dnaK
dacA
sdhA
edd
PP_2347
PP_0897
purH
atpA
metK
glyA-2
pnp
rpsA
atpD
ilvC
purB
lpdG
leuC
ndk
serA
leuB
PP_4867
glnA
accC-2
PP_0412
metF
gdhA
ohr
katG
oprF
oprD
glnK
gltI
PP_0998
PP_0861
argB
argC1
rmlB
B)
aapJ
PP_1726
PP_2347
PP_1689
PP_0525
PP_0998
PP_0765
PP_0412
ohr
katG
accC-1
bktB
arcA
PP_2132
fda
proC-1
ygaU
metK
pgl
dacA
tsf
tuf-2
sucC
PP_0897
gap-1
purH
rplL
groEL
tuf-1
glyA-2
edd
pnp
ilvC
purB
lpdG
dnaK
atpD
rpsA
leuB
accC-2
fliC
atpA
PP_4867
PP_0861
gdhA
leuC
glnA
htpG
serA
oprF
oprD
syrB
PP_3787
PP_1661
PP_1291
gltI
thiG
glnK
